# Novel *OPN1LW/OPN1MW* Exon 3 Haplotype-Associated Splicing Defect in Patients with X-Linked Cone Dysfunction

**DOI:** 10.3390/ijms23126868

**Published:** 2022-06-20

**Authors:** Katarina Stingl, Britta Baumann, Pietro De Angeli, Ajoy Vincent, Elise Héon, Monique Cordonnier, Elfriede De Baere, Salmo Raskin, Mario Teruo Sato, Naoye Shiokawa, Susanne Kohl, Bernd Wissinger

**Affiliations:** 1 Centre for Ophthalmology, University Eye Hospital, University of Tübingen, 72076 Tübingen, Germany; katarina.stingl@med.uni-tuebingen.de; 2 Institute for Ophthalmic Research, Centre for Ophthalmology, University of Tübingen, 72076 Tübingen, Germany; britta.baumann@med.uni-tuebingen.de (B.B.); pietro.de-angeli@med.uni-tuebingen.de (P.D.A.); susanne.kohl@med.uni-tuebingen.de (S.K.); 3 Department of Ophthalmology and Vision Sciences, The Hospital for Sick Children and University of Toronto, Toronto, ON M5G 1X8, Canada; ajoy.vincent@sickkids.ca (A.V.); elise.heon@sickkids.ca (E.H.); 4 Department of Ophthalmology, Hôpital Erasme, Cliniques Universitaires de Bruxelles, Université Libre de Bruxelles, 1070 Bruxelles, Belgium; mcordonn@skynet.be; 5 Center for Medical Genetics, Ghent University Hospital, Department of Biomolecular Medicine, Ghent University, 9000 Ghent, Belgium; elfride.debaere@ugent.be; 6 Laboratório Genetika, Curitiba 80730-180, Brazil; s.raskin@genetika.com.br; 7 Department of Ophthalmology & Otorhinolaryngology, Federal University of Paraná, Curitiba 80060-900, Brazil; sato.mario@gmail.com; 8 Retina and Vitreo Consulting Eye Clinic, Curitiba 80530-010, Brazil; naoyeshiokawa@yahoo.com.br

**Keywords:** cone photoreceptor LWS and MWS opsin genes, haplotype, exonic splicing defect, minigene assay, Blue Cone Monochromacy, Bornholm Eye Disease

## Abstract

Certain combinations of common variants in exon 3 of *OPN1LW* and *OPN1MW*, the genes encoding the apo-protein of the long- and middle-wavelength sensitive cone photoreceptor visual pigments in humans, induce splicing defects and have been associated with dyschromatopsia and cone dysfunction syndromes. Here we report the identification of a novel exon 3 haplotype, G-C-G-A-T-T-G-G (referring to nucleotide variants at cDNA positions c.453, c.457, c.465, c.511, c.513, c.521, c.532, and c.538) deduced to encode a pigment with the amino acid residues L-I-V-V-A at positions p.153, p.171, p.174, p.178, and p.180, in *OPN1LW* or *OPN1MW* or both in a series of seven patients from four families with cone dysfunction. Applying minigene assays for all observed exon 3 haplotypes in the patients, we demonstrated that the novel exon 3 haplotype L-I-V-V-A induces a strong but incomplete splicing defect with 3–5% of residual correctly spliced transcripts. Minigene splicing outcomes were similar in HEK293 cells and the human retinoblastoma cell line WERI-Rb1, the latter retaining a cone photoreceptor expression profile including endogenous *OPN1LW* and *OPN1MW* gene expression. Patients carrying the novel L-I-V-V-A haplotype presented with a mild form of Blue Cone Monochromacy or Bornholm Eye Disease-like phenotype with reduced visual acuity, reduced cone electroretinography responses, red-green color vision defects, and frequently with severe myopia.

## 1. Introduction

*OPN1LW* and *OPN1MW* encode the apo-proteins of the long-wavelength-sensitive (LWS) and middle-wavelength-sensitive (MWS) cone photopigments, respectively. *OPN1LW* and *OPN1MW* share high sequence homology and are arranged as a tandem repeat gene cluster on Xq28 with a single *OPN1LW* gene followed by one or multiple *OPN1MW* gene copies [1]. Their expression is governed by the locus control region (LCR), an upstream enhancer element that selectively contacts the proximal promoter of *OPN1LW* or *OPN1MW* to drive mutual exclusive expression of the one or the other gene in individual cone photoreceptors [2].

Structural rearrangements and mutations in the *OPN1LW*/*OPN1MW* gene cluster underlie common forms of red-green colorblindness (protan and deutan deficiencies) as well as several rare cone dysfunction disorders such as Blue Cone Monochromacy (BCM), X-linked Cone Dystrophy (Xl-CD) and Bornholm Eye Disease (BED) [3,4,5,6].

In 2012, Ueyama and colleagues showed that a small fraction of protanopic and protanomalous color vision defects are linked to rare combinations of exon 3 single nucleotide polymorphisms in *OPN1LW* which induces mis-splicing and skipping of exon 3 [7]. Ever since, we and others have corroborated and extended exon 3 haplotype-induced splicing defects of *OPN1LW* and *OPN1MW* genes as a frequent cause of cone dysfunction disorders in patients with BCM, Xl-CD and BED [8,9,10,11,12].

So far, only a few distinct exon 3 haplotypes have been observed recurrently in patients and validated by in vitro splicing assays including G-C-G-A-T-C-G-G (c.(453A > G; 457A > C; 465C > G; 511G > A; 513G > T; 521C; 532A > G; 538T > G), frequently denoted as ‘L-I-A-V-A’, from the therefrom deduced amino acid residues (p.[(=); M153L; (=); V171I; A174A; I178V; S180A]), G-C-G-G-G-C-G-G (‘L-V-A-V-A’), G-C-G-A-T-C-G-T (‘L-I-A-V-S’) and A-A-G/C-A-T-C-G-G (‘M-I-A-V-A’). The assessment of other rare exon 3 haplotypes is still lacking.

Here we present clinical and functional analysis data which demonstrate that G-C-G-A-T-T-G-G (‘L-I-V-V-A’) is another deleterious exon 3 haplotype that induces exon 3 skipping and is associated with cone dysfunction disorders in seven male patients from four families.

## 2. Results

### 2.1. Identification of Patients with the Novel LIVVA Exon 3 Haplotype in the OPN1LW and/or the OPN1MW Gene and Functional Minigene Splicing Assays

**Figure 1:**
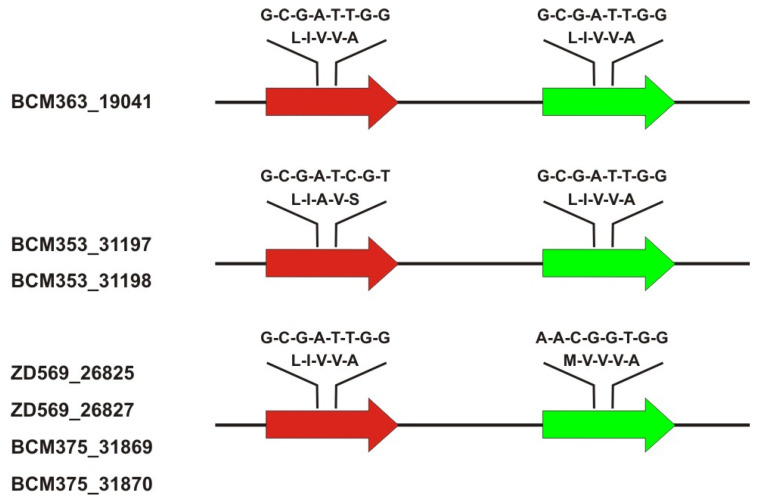
*OPN1LW* and *OPN1MW* exon 3 genotypes in the study group. Overview of the *OPN1LW*/*OPN1MW* gene cluster composition and exon 3 haplotypes in the investigated patients. Exon 3 haplotypes are depicted at the nucleotide sequence level (cDNA sequence positions: c.453-c.457-c.465-c.511-c.513-c.521-c.532-c.538) and the corresponding amino acid residues (at positions p.153-p.171-p.174-p.178-p.180).

The common denominator of the probands in this study is the presence of a novel rare exon 3 haplotype G-C-G-A-T-T-G-G (‘L-I-V-V-A’) in one or all copies of the X-linked cone opsin genes (Table 1, Figure 1). All probands had a structurally normal *OPN1LW*/*OPN1MW* gene cluster with a single *OPN1LW* and a single *OPN1MW* gene copy.

The novel G-C-G-A-T-T-G-G (‘L-I-V-V-A’) haplotype was present either in both *OPN1LW* and *OPN1MW* (BCM363_19041), only in the *OPN1LW* gene (ZD569 and BCM375 family, two affected brothers each) or only in the *OPN1MW* gene (both brothers of the BCM353 family). Both brother pairs in the ZD569 and the BCM375 families carried the A-A-C-G-G-T-G-G (‘M-V-V-V-A’) exon 3 haplotype in their *OPN1MW* gene copy, and both brothers of the BCM353 family carried the G-C-G-A-T-C-G-T (‘L-I-A-V-S’) exon 3 haplotype in their *OPN1LW* gene (Table 1, Figure 1).

**Figure 2:**
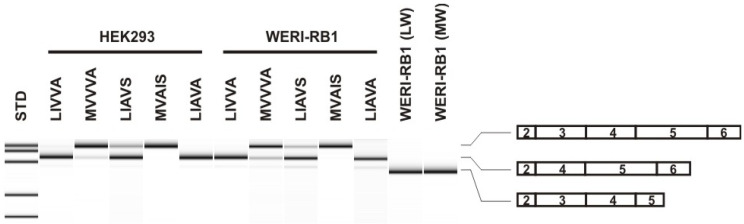
Bioanalyzer separation of RT-PCRs from HEK293 and WERI-Rb1 cells. RT-PCR products obtained with RNA from HEK293 and WERI-Rb1 cells transfected with different exon 3 minigene constructs were separated on a Bioanalyzer DNA-1000 chip. The exonic composition of the products is indicated on the right. RT-PCR products with RNA from non-transfected WERI-Rb1 cells showing expression and correct splicing of endogenous *OPN1LW* and *OPN1MW* transcripts were separated in comparison (lanes WERI-Rb1 (LW) and WERI-Rb1 (MW)). Note that different reverse primers were used for amplification of cDNA from minigene transfected cells (i.e., construct-specific reverse primers) and non-transfected WERI-Rb1 cells, which also results in fragment size differences. STD—Size Standard.

We used an established minigene assay to analyze the effect of the novel G-C-G-A-T-T-G-G (‘L-I-V-V-A’) exon 3 haplotype in HEK293 cells. Assays were also performed in parallel for the two other exon 3 haplotypes observed in the probands as well as two control haplotypes, G-C-G-A-T-C-G-G (‘LIAVA’) and A-A-C-G-G-C-A-T (‘MVAIS’). In HEK293 cells, we observed a strong, near-complete splicing defect of the novel G-C-G-A-T-T-G-G (‘L-I-V-V-A’) haplotype with only 5.28 ± 0.09% of correctly spliced transcripts (Table 2, Figure 2). The fractions of correctly spliced transcripts for the other haplotypes were: 28.36 ± 0.5% for G-C-G-A-T-C-G-T (‘L-I-A-V-S’), 74.41 ± 0.5% for A-A-C-G-G-T-G-G (‘M-V-V-V-A’), and for the controls: non-detectable levels of correctly spliced transcripts for G-C-G-A-T-C-G-G (‘LIAVA’) and 98.12 ± 0.07% for A-A-C-G-G-C-A-T (‘MVAIS’), values which are similar to previous reports [7,8,9,10]. The results of the minigene assay in HEK293 cells suggest a strong deleterious effect on exon 3 splicing for the novel haplotype G-C-G-A-T-T-G-G (‘L-I-V-V-A’).

We further investigated the reliability of the minigene assay in HEK293 cells in comparison with WERI-Rb1, a retinoblastoma cell line with cone-like character and expression profile [13,14]. In fact, we found that WERI-Rb1 cells well express *OPN1LW* (exon 3 haplotype: A-A-C-G-G-C-A-T [‘MVAIS’]) and *OPN1MW* (exon 3 haplotype: A-A-C-G-G-C-A-G [‘MVAIA’]) endogenously with no evidence of aberrantly spliced transcripts (Figure 2). We found that the splicing outcome of minigene constructs expressed in WERI-Rb1 cells was highly similar to that observed in HEK293 cells (Table 2), confirming that HEK293 cells are well suited for the analysis of *OPN1LW* and *OPN1MW* exon 3 splice defects.

### 2.2. Clinical Findings

Seven male patients were examined in this project, and all but one (BCM363_19041) had at least one follow-up examination (Table 3). The youngest patient was 4 years old, and the oldest at follow-up was 15 years old. The age of onset of the first visual symptoms varied between 3 years and 10 years. In two patients, the age of onset was unknown as they did not perceive any subjective visual difficulties prior to eye examinations. For the rest of the patients, decreased BCVA was the leading first symptom. One patient (BCM363_19041) reported dyschromatopsia in ambient bright light conditions.

The BCVA of all patients was usually decreased to the middle range, but varied between 20/125 and 20/20. There was no clear difference between the BCVA values of patients with the different *OPN1LW* and *OPN1MW* exon 3 haplotype combinations; however, full visual acuity was only reached by ZD569_26825, an almost asymptomatic patient carrying the novel LIVVA exon 3 haplotype in *OPN1LW* and the common MVVVA exon 3 haplotype in *OPN1MW*. All but two patients (BCM363_19041 who was emmetropic and BCM353_31197 who was hyperopic at the first visit at the age of 4 years and emmetropic at the follow-up visit at the age of 10 years) had high myopia up to −20 spherical diopters with astigmatism. Five patients reported photophobia, only two (BCM353_31197 and _31198) did not. If color vision tests were performed, the majority of the patients had mixed confusions along the red-green axes, but in two of the patients carrying the LIVVA exon 3 haplotype in *OPN1MW* pure green-color deficits were recorded. The borders of visual fields were normal in all subjects.

In full-field electroretinography (ERG), all patients showed normal scotopic recordings confirming well-functioning rods. Photopic recordings, however, showed various degrees of reduced cone functionality with reduced amplitudes and some prolonged implicit times, without clear differences in cone-driven recordings between patients with the LIVVA exon 3 haplotype in *OPN1MW* or *OPN1LW*.

**Figure 3:**
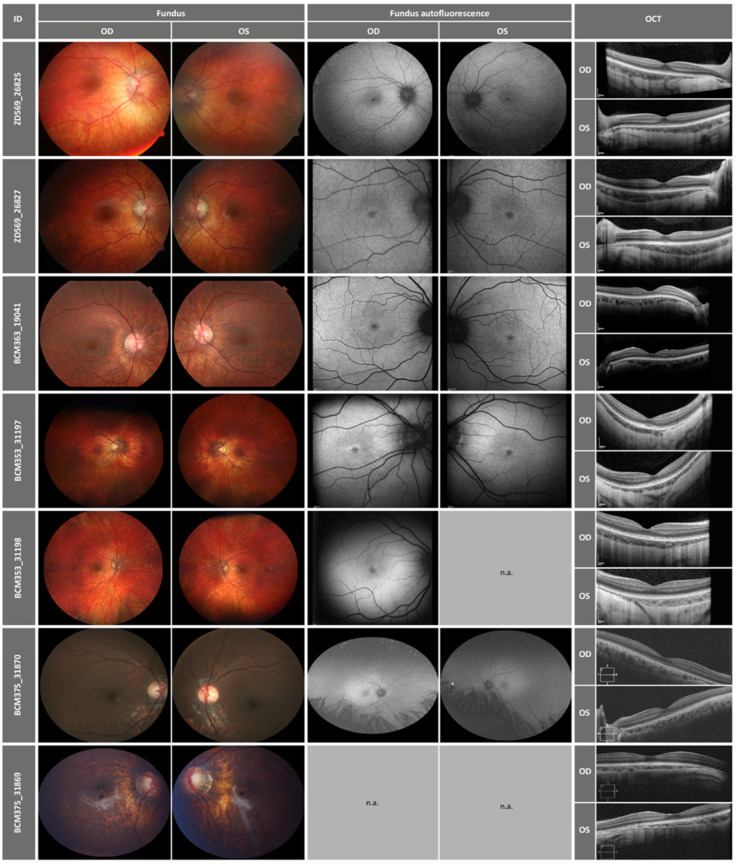
Retinal imaging of the patients with fundus and fundus autofluorescence photography, as well as OCT images. OD = right eye, OS = left eye.

Retinal imaging was almost normal, but all patients had a rather hypopigmented fundus appearance with myopic peripapillary atrophy in particular in the myopic patients, as well as some slight temporal paleness of the optic nerve head (Figure 3). Although OCTs showed normal layering of the central retina, all patients with the LIVVA exon 3 haplotype in *OPN1MW* had slight signs of diffuse disruption of the central outer segments. Additionally, patients BCM375_31869 and BCM375_31870 showed a very mild form of foveal hypoplasia in FAF. Decent foveal and perifoveal changes were observed in the patient cohort in FAF, which were characterized by a hypopigmented fovea with usually a slight hyperpigmentation perifoveal ring. An overview of the clinical findings is given in the Figure 3 and Table 3.

## 3. Discussion

Exon 3 haplotype-induced splicing deficiency comprises a rather new category of molecular pathology in *OPN1LW*/*OPN1MW* opsin gene cluster linked disease. So far, only a small number of unequivocally deleterious exon 3 haplotypes have been recurrently reported and their splicing deficiency functionally confirmed by minigene assays ([7,8,9,10]. Herein, we report a novel splicing-deficient exon 3 haplotype, G-C-G-A-T-T-G-G (‘L-I-V-V-A’), observed in a number of male probands with cone dysfunction disorders. The minigene assay confirmed a severe splicing deficiency with only about 5.4% and 3.9% of correctly spliced transcripts in HEK293 and WERI-Rb1 cells, respectively. This fraction is higher than in the prototypical splicing-deficient ‘L-I-A-V-A’ haplotype but lower than for the ‘L-I-A-V-S’ haplotype first described by Mizrahi-Meissonier and colleagues [5] and also found in the *OPN1LW* gene copy in the two brothers of the BCM353 family. To the best of our knowledge, our study for the first time investigated the exon 3 haplotype-linked splicing defect in WERI-Rb1, a retinoblastoma cell line with cone-like character and expression profile. In fact, we demonstrated endogenous *OPN1LW* and *OPN1MW* gene expression and its proper splicing in WERI-Rb1 cells (Figure 2). The results obtained with the WERI-Rb1 cell line closely resemble and support the reliability—also in quantitative terms—of the HEK293 cell system for the assessment of *OPN1LW* and *OPN1MW* exon 3 linked splicing defects.

The clinical phenotype of the patients reported here can be described as BCM or BED. BED has been described as represented by X-linked myopia with astigmatism, deuteranopia and reduced cone responses with hypoplasia of the optic nerve head [15,16,17,18]. BCM is a congenital X-chromosomal functional disorder of both red and green cones [19,20].

Although the phenotypes of BED and BCM overlap, optic nerve head atrophy, high myopia and better preserved red color perception are rather characteristic of BED. The cohort described here had a fundus appearance compatible with BED with slight temporal paleness of the optic nerve head and hypopigmented fundi. Retinal imaging with OCT showed a slight diffuse disruption of the central photoreceptor outer segments in patients carrying the LIVVA exon 3 haplotype in *OPN1MW*, but these changes were rather discreet. The remaining patients had a rather normal foveal morphology (ZD569_26825 and _26827) or a small level of foveal hypoplasia (BCM535_31197 and _31198).

The functional aspects of the vision in our cohort did not allow for a precise differentiation into the categories of BED or BCM based on exon 3 haplotypes. Still, a pure green-color defect was observed only in two patients carrying the LIVVA haplotype in *OPN1MW* (BCM363_19041 and BCM353_31198). Although this would be characteristic for BED, exactly these two patients were emmetropic, thus not fulfilling the high myopia criterion of BED. Additionally, in hue tests, the green and red color axes are similar to each other, thus a pure green-color defect detected by hue tests might be to some part also mixed with red-color vision problems. All other patients had a color vision defect of both red (L) and green (M) cones. Rod functionality, measured with full-field ERG, seemed normal in the whole cohort. This is in contrast with the previously published phenotype of exon 3 opsin phenotypes described as X-linked myopia with normal visual acuity, color perception and rod affection [21].

In our cohort, cones were affected to different extents; however, color disturbance or some degree of BCVA reduction and reduced photopic ERG were present in all patients. However, there were two patients who did not report subjective symptoms before ophthalmologic examination. Otherwise, the cone functional loss in the sense of decreased visual acuity at preschool age or early school age was the leading symptom. These findings are in concordance with BED [22] but also BCM.

Because our cohort included only minors, we cannot report clinical progression of the cones’ functional loss into adulthood. Still, the follow-ups in these patients covering preschool age and teenage confirmed rather stable findings consistent with BCM in general and corresponded with previously reported findings [23].

Morphological changes of the retinas in these patients were rather decent. Close to normal retinal layering was observed in OCT images and retinal appearance showed some degree of hypopigmentation and optic nerve head paleness. Because many of the patients had myopia, myopic changes such as peripapillary atrophy were present, too. Findings of macular atrophy, such as reported in affected adults by Orosz and colleagues [21], were not observed in our cohort. There were only small changes in the perifoveal autofluorescence in our patients, reflecting probably a small degree of pigment epithelium changes in the area of the highest red (L) and green (M) cone density.

In summary, the phenotype of this cohort represents a BCM phenotype with various degrees of color perception problems, mostly with myopia, astigmatism and slight optic nerve head atrophy, sharing many features with the BED phenotype [6] although none of our patients fulfilled all typical BED criteria, i.e., a deutan color vision defect, high myopia and optic nerve head atrophy. A clear phenotypic distinction between subjects with the L-I-V-V-A exon 3 haplotype either in *OPN1LW* or in *OPN1MW* was not observed in our small patient cohort.

## 4. Materials and Methods

### 4.1. Patient Recruitment and Clinical Evaluation

The study was conducted in accordance with the tenets of the World Medical Association Declaration of Helsinki. Patients were recruited as part of local clinical studies or ad hoc at different centers specialized in inherited retinal diseases during routine clinical diagnostics. Blood samples for genetic analysis were obtained from all patients upon written informed consent. The study was approved by the institutional review board of the Ethics Committee of the University Hospital of Tübingen (project No. 116/2015BO2, last update and review 9 February 2021).

Patients underwent multimodal clinical examinations including functional tests of the retina as well as retinal imaging. All patients were asked for detailed ophthalmological history with age of first symptom onset, presence of night blindness and photophobia. Examinations included best corrected visual acuity (BCVA), color vision testing using mostly the Farnsworth 15 hue test, full-field electroretinography and kinetic perimetry or gross confrontational perimetry in children. Retinal imaging with fundus photography, spectral domain optical coherence tomography (Spectralis OCT) and fundus autofluorescence (FAF, both with Heidelberg Engineering, Heidelberg, Germany) were also performed.

### 4.2. Genotyping of the OPN1LW/OPN1MW Gene Cluster

The integrity and composition of the *OPN1LW*/*OPN1MW* gene cluster was analyzed by a custom screening protocol as described previously [24]. Long-distance PCR was used to specifically amplify parts of the *OPN1LW* gene and *OPN1MW* gene, respectively, and to determine the exon 3 sequences by Sanger sequencing.

Multiplex ligation-dependent probe amplification (MLPA) was used to determine the copy number at the *OPN1LW*/*OPN1MW* gene cluster. For MLPA we used a premarketing release version of the SALSA X080-B1 Opsin probe-mix (MRC Holland, Amsterdam, The Netherlands) targeting sequences of the LCR and individual exons of *OPN1LW* and *OPN1MW* specifically. MLPA was performed according to the manufacturer’s recommendations and amplification products were separated by capillary electrophoresis on an ABI 3130XL (Applied Biosystems/ThermoFisher Scientific, Weiterstadt, Germany) instrument. Data analysis was performed using the Coffalyser.Net software (MRC Holland, Amsterdam, The Netherlands) which calculates ratios in comparison to reference samples co-processed with the test samples.

### 4.3. Genotyping of the OPN1LW/OPN1MW Gene Cluster

The prototype *OPN1LW* opsin minigene construct was kindly provided by Dr. Ueyama (Shiga University, Otsu, Japan) and used to generate minigene constructs with *OPN1LW* or *OPN1MW* gene variant combinations in exon 3, flanked by its full-length upstream and downstream introns and the remaining human *OPN1LW* cDNA [9]. In brief, a fragment encompassing exon 3 and parts of the flanking introns were PCR-amplified from genomic DNA from probands and inserted into the minigene vector backbone by conventional cloning procedures. The inserted sequences were verified by Sanger sequencing.

Minigene constructs were heterologously expressed in HEK293 cells and WERI-Rb1 cells [13]. HEK293 cells were transfected using Lipofectamine 2000 (Invitrogen—Thermo Fisher Scientific, Dreieich, Germany), whereas electroporation using the following setting: 1400V, 10ms, 2 pulses (Neon Electroporation System, ThermoFisher Scientific, Weiterstadt, Germany) was used to deliver plasmid DNA to WERI-Rb1 cells. Total RNA was isolated 24–48 h post-transfection using the peqGold Total RNA Isolation Kit (PeqLab Biotechnologie GmbH, Erlangen, Germany). One microgram of total RNA was used for reverse transcription using random hexamers (7BioScience, Neuenburg, Germany). Subsequent PCR was performed with a 5′ FAM-labeled forward primer FEO35 (5′-ACCATGAAGTTCAAGAAGCT-3′) and a non-labeled reverse primer IFC-inv-BC-RV (5′-CTTGTCATCGTCGTCCTTGTAA-3′). PCR products were separated on an ABI 3130XL capillary electrophoresis instrument and the area-under-the-curve (AUC) values used to determine the fraction of correctly spliced transcripts. Minigene assays in HEK293 cells were performed in triplicates. RT-PCR products were also separated on an Agilent Bioanalyzer 2100 (Agilent, Waldbronn, Germany) using a DNA 1000 Labchip and reagents. Endogenously expressed *OPN1LW* and *OPN1MW* mRNA was amplified from reverse-transcribed cDNA using CB79R (5′-CCAGCAGACGCAGTACGCAAAGATC-3′) and CB79G (5′- CCAGCAGAAGCAGAATGCCAGGAC-3′), respectively, as reverse primers.

## Figures and Tables

**Table 1:** *OPN1LW* and *OPN1MW* exon 3 genotypes in the study group.

Subject/Proband	*OPN1LW* Exon 3 Haplotype (nt/aa) ^1^		*OPN1MW* Exon 3 Haplotype (nt/aa) ^1^		∑ Gene Copies ^2^
BCM363_19041	G-C-G-A-T-T-G-G	L-I-V-V-A	G-C-G-A-T-T-G-G	L-I-V-V-A	2
BCM353_31197	G-C-G-A-T-C-G-T	L-I-A-V-S	G-C-G-A-T-T-G-G	L-I-V-V-A	2
BCM353_31198	G-C-G-A-T-C-G-T	L-I-A-V-S	G-C-G-A-T-T-G-G	L-I-V-V-A	2
ZD569_26825	G-C-G-A-T-T-G-G	L-I-V-V-A	A-A-C-G-G-T-G-G	M-V-V-V-A	2
ZD569_26827	G-C-G-A-T-T-G-G	L-I-V-V-A	A-A-C-G-G-T-G-G	M-V-V-V-A	2
BCM375_31869	G-C-G-A-T-T-G-G	L-I-V-V-A	A-A-C-G-G-T-G-G	M-V-V-V-A	2
BCM375_31870	G-C-G-A-T-T-G-G	L-I-V-V-A	A-A-C-G-G-T-G-G	M-V-V-V-A	2

^1^ nt—nucleotides at c.453, c.457, c.465, c.511, c.513, c.521, c.532, and c.538 (references: NM_020061.6 and NM_000513.2), aa—amino acids at p.153, p.171, p.174, p.178, and p.180; ^2^ sum of *OPN1LW* and *OPN1MW* gene copies.

**Table 2:** Splicing of *OPN1LW* and *OPN1MW* exon 3 haplotype minigene constructs in HEK293 and WERI-Rb1 cells.

Exon 3 Haplotype (nt/aa)		% Correctly Spliced	
		HEK293 ^1^	WERI-Rb1 ^1^
G-C-G-A-T-T-G-G	L-I-V-V-A	5.28 ± 0.088	3.89 ± 0.18
G-C-G-A-T-C-G-T	L-I-A-V-S	28.36 ± 0.5	18.21 ± 0.24
A-A-C-G-G-T-G-G	M-V-V-V-A	74.41 ± 0.5	69.44 ± 0.17
G-C-G-A-T-C-G-G	L-I-A-V-A	0.00/ndt.	0.00/ndt.
A-A-C-G-G-C-A-T	M-V-A-I-S	98.12 ± 0.07	98.22 ± 0.16

^1^ ndt.—non-detectable.

**Table 3:** Clinical findings including medical history of all patients.

Patient-ID	Age at Onset ^1^	First Symptom	Age (Years) ^1^	BCVA ^2^ (Decimal)		Refraction ^2^		Night Blindness	Photo- Phobia	Full-Field ERG	Color Test (Farnsworth 15 Hues)	Visual Field
				OD	OS	OD	OS					
ZD569 _26825	not known	no symptoms	10	1.0	0.63	−1.25/−2.5/151	−0.5/−2.0/180	no	yes	sctotopic normal, photopic very reduced	scotopic, red and green color confusions	III4e borders normal
			13	1.0	0.8	−7.75/−3.25/21	−7.0/−33.5/98	no	yes	photopic very reduced	n.a.	III4e borders normal
ZD569 _26827	6	decreased visual acuity	8	0.4	0.5	−7.5/−4.25/29	−7.0/−3.75/174	no	yes	sctotopic normal, photopic very reduced	red and green color confusions	III4e borders normal
			11	0.8	0.6	−9.5/−4.25/23	−9.25/−3.25/160	no	yes	sctotopic normal, photopic very reduced	n.a.	III4e borders normal
BCM363 _19041	10	dyschromatspia in brightness	14	0.4	0.4	emmetropia		no	yes	sctotopic normal, photopic very reduced	green color confusions	III4e borders normal
BCM353 _31197	3	decreased visual acuity	6	0.3	0.4	−9/−2.5/15	−9.5/−1/20	no	no	photopic and flicker reduced, prolonged flicker implicit times	red and green color confusions (desatured test only)	Grossly normal (confrontation perimetry)
			11	0.4	0.5	−7/−2/140	−9/−1/145	no	no	n.a.	red and green color confusions (saturated and desatured test)	relative central scotoma (automated perimetry)
BCM353 _31198	not known	no symptoms	4	0.4	0.4	+5.5/−1/15	+4.25/−2/175	no	no	normal except flicker: reduced and prolonged implicit time	n.a.	n.a.
			10	0.8	0.6	emmetropia		no	no	n.a.	green-color deficits (Ishihara)	n.a.
BCM375 _31870	4	decreased visual acuity	9	0.63	0.8	−9.75	−8.00/+1.00/90	no	mild hemeralopia	scotopic normal, photopic reduced	strong red and green color deficits	well preserved
			15	0.5	0.8	−11.50/+1.25/25	−9.75/+1.00/95	no	mild photophobia	n.a.	strong red green color deficits	n.a.
BCM375 _31869	3	decreased visual acuity	7	0.25	0.16	−20.00/+1.75/125	−20.00/+2.00/90	no	yes	scotopic normal, photopic severyl reduced	moderate red and green color deficits	well preserved
			14	0.5	0.2	−23.00/+3.00/100	−24.00/+1.00/90	no	yes	n.a.	moderate red and green color deficits	n.a.

^1^ age of onset as well as age of examination is given in years. ^2^ BCVA (best corrected visual acuity) values are decimal, OD = right eye, OS = left eye, n.a. = data not available.

## Data Availability

All analyzed data are included in the manuscript. Raw data may be obtained from the authors upon written request.

## References

[1] Nathans J., Thomas D., Hogness D.S. (1986). Molecular genetics of human color vision: The genes encoding blue, green, and red pigments. Science.

[2] Smallwood P.M., Wang Y., Nathans J. (2002). Role of a locus control region in the mutually exclusive expression of human red and green cone pigment genes. Proc. Natl. Acad. Sci. USA.

[3] Nathans J., Piantanida T.P., Eddy R.L., Shows T.B., Hogness D.S. (1986). Molecular genetics of inherited variation in human color vision. Science.

[4] Nathans J., Davenport C.M., Maumenee I.H., Lewis R.A., Hejtmancik J.F., Litt M., Lovrien E., Weleber R., Bachynski B., Zwas F. (1989). Molecular genetics of human blue cone monochromacy. Science.

[5] Mizrahi-Meissonnier L., Merin S., Banin E., Sharon D. (2010). Variable retinal phenotypes caused by mutations in the X-linked photopigment gene array. Investig. Ophthalmol. Vis. Sci..

[6] Michaelides M., Johnson S., Bradshaw K., Holder G.E., Simunovic M.P., Mollon J.D., Moore A.T., Hunt D.M. (2005). X-linked cone dysfunction syndrome with myopia and protanopia. Ophthalmology.

[7] Ueyama H., Muraki-Oda S., Yamade S., Tanabe S., Yamashita T., Shichida Y., Ogita H. (2012). Unique haplotype in exon 3 of cone opsin mRNA affects splicing of its precursor, leading to congenital color vision defect. Biochem. Biophys. Res. Commun..

[8] Gardner J.C., Liew G., Quan Y.-H., Ermetal B., Ueyama H., Davidson A.E., Schwarz N., Kanuga N., Chana R., Maher E.R. (2014). Three Different Cone Opsin Gene Array Mutational Mechanisms with Genotype–Phenotype Correlation and Functional Investigation of Cone Opsin Variants. Hum. Mutat..

[9] Buena-Atienza E., Rüther K., Baumann B., Bergholz R., Birch D., Baere E.D., Dollfus H., Greally M.T., Gustavsson P., Hamel C.P. (2016). De novo intrachromosomal gene conversion from OPN1MW to OPN1LW in the male germline results in Blue Cone Monochromacy. Sci. Rep..

[10] Greenwald S.H., Kuchenbecker J.A., Rowlan J.S., Neitz J., Neitz M. (2017). Role of a Dual Splicing and Amino Acid Code in Myopia, Cone Dysfunction and Cone Dystrophy Associated with L/M Opsin Interchange Mutations. Transl. Vis. Sci. Technol..

[11] Holmquist D., Epstein D., Olsson M., Wissinger B., Kohl S., Hengstler J., Tear-Fahnehjelm K. (2021). Visual and ocular findings in a family with X-linked cone dysfunction and protanopia. Ophthalmic Genet..

[12] Khateb S., Shemesh A., Offenheim A., Sheffer R., Ben-Yosef T., Chowers I., Leibu R., Baumann B., Wissinger B., Kohl S. (2022). Relatively mild blue cone monochromacy phenotype caused by various haplotypes in the L- and M-cone opsin genes. Mol. Vis..

[13] McFall R.C., Sery T.W., Makadon M. (1977). Characterization of a new continuous cell line derived from a human retinoblastoma. Cancer Res..

[14] Shaaban S.A., Deeb S.S. (1998). Functional analysis of the promoters of the human red and green visual pigment genes. Investig. Ophthalmol. Vis. Sci..

[15] Patterson E.J., Wilk M., Langlo C.S., Kasilian M., Ring M., Hufnagel R.B., Dubis A.M., Tee J.J., Kalitzeos A., Gardner J.C. (2016). Cone Photoreceptor Structure in Patients With X-Linked Cone Dysfunction and Red-Green Color Vision Deficiency. Investig. Ophthalmol. Vis. Sci..

[16] McClements M., Davies W.I., Michaelides M., Young T., Neitz M., MacLaren R.E., Moore A.T., Hunt D.M. (2013). Variations in opsin coding sequences cause x-linked cone dysfunction syndrome with myopia and dichromacy. Investig. Ophthalmol. Vis. Sci..

[17] Haim M., Fledelius H.C., Skarsholm D. (1988). X-linked myopia in Danish family. Acta Ophthalmol..

[18] Schwartz M., Haim M., Skarsholm D. (1990). X-linked myopia: Bornholm eye disease. Linkage to DNA markers on the distal part of Xq. Clin. Genet..

[19] Blackwell H.R., Blackwell O.M. (1957). Blue mono-cone monochromacy: A new color vision defect. J. Opt. Soc. Am..

[20] Alpern A., Lee G.B., Spivey B.E. (1965). π1 cone monochromatism. Arch. Ophthalmol..

[21] Orosz O., Rajta I., Vajas A., Takács L., Csutak A., Fodor M., Kolozsvári B., Resch M., Sényi K., Lesch B. (2017). Myopia and Late-Onset Progressive Cone Dystrophy Associate to LVAVA/MVAVA Exon 3 Interchange Haplotypes of Opsin Genes on Chromosome X. Investig. Ophthalmol. Vis. Sci..

[22] Young T.L., Deeb S.S., Ronan S.M., Dewan A.T., Alvear A.B., Scavello G.S., Paluru P.C., Brott M.S., Hayashi T., Holleschau A.M. (2004). X-linked high myopia associated with cone dysfunction. Arch. Ophthalmol..

[23] Patterson E.J., Kalitzeos A., Kasilian M., Gardner J.C., Neitz J., Hardcastle A.J., Neitz M., Carroll J., Michaelides M. (2018). Residual Cone Structure in Patients With X-Linked Cone Opsin Mutations. Investig. Ophthalmol. Vis. Sci..

[24] Cideciyan A.V., Hufnagel R.B., Carroll J., Sumaroka A., Luo X., Schwartz S.B., Dubra A., Land M., Michaelides M., Gardner J.C. (2013). Human cone visual pigment deletions spare sufficient photoreceptors to warrant gene therapy. Hum. Gene Ther..

